# High blood pressure in school children: prevalence and risk factors

**DOI:** 10.1186/1471-2431-6-32

**Published:** 2006-11-16

**Authors:** Ximena Urrutia-Rojas, Christie U Egbuchunam, Sejong Bae, John Menchaca, Manuel Bayona, Patrick A Rivers, Karan P Singh

**Affiliations:** 1 Department of Social and Health Behavior, School of Public Health, University of North Texas Health Science Center, Fort Worth, Texas, USA; 2 Department of Biostatistics, School of Public Health, University of North Texas Health Science Center, Fort Worth, Texas, USA; 3 Pediatrics, Cook Children's Hospital Network, Dallas, Texas, USA; 4 Department of Epidemiology, School of Public Health, University of North Texas Health Science Center, Fort Worth, Texas, USA; 5 Health Care Management, Southern Illinois University, Carbondale, Illinois, USA

## Abstract

**Background:**

The purpose of this study was to determine the prevalence of high blood pressure (HBP) and associated risk factors in school children 8 to 13 years of age.

**Methods:**

Elementary school children (n = 1,066) were examined. Associations between HBP, body mass index (BMI), gender, ethnicity, and acanthosis nigricans (AN) were investigated using a school based cross-sectional study. Blood pressure was measured and the 95^th ^percentile was used to determine HBP. Comparisons between children with and without HBP were utilized. The crude and multiple logistic regression adjusted odds ratios were used as measures of association.

**Results:**

Females, Hispanics, overweight children, and children with AN had an increased likelihood of HBP. Overweight children (BMI ≥ 85^th ^percentile) and those with AN were at least twice as likely to present with HBP after controlling for confounding factors.

**Conclusion:**

Twenty one percent of school children had HBP, especially the prevalence was higher among the overweight and Hispanic group. The association identified here can be used as independent markers for increased likelihood of HBP in children.

## Background

In 2002, the National Center for Health Statistics revealed that the prevalence of hypertension was 28.7% among Americans 20–74 years of age; and that 84.9% of women and 72.7% of men 75 years of age and older have hypertension[1]. The prevalence of hypertension in African Americans 20 years of age and older, was 40.9% and 37.8% for females and males, respectively. In Caucasians of the same age group, the prevalence was 24.5% for females and 28.8% for males. In Hispanics of this same age group, the prevalence of hypertension was 25% for females and 30.6% for males[1]. Over the past two decades, studies have shown that "essential" hypertension (i.e., hypertension of unknown etiology), can be found among children and adolescents. These particular blood pressure (BP) patterns show a strong correlation to adulthood hypertension [2-5]. According to the recommendations of the 1996 task force report on BP in children and adolescents, BP measurements should be incorporated into the routine pediatric examination of children three years of age and older[2]. Although the prevalence of hypertension during childhood is lower than that seen in adulthood, this condition is not rare in children, thus stressing the importance of evaluating BP [6]. The prevalence of hypertension among children reported by various studies ranges from 5.4% to 19.4% [7-9,11].

Factors known to affect BP among children include age, sex, body size, race/ethnicity, obesity, and socioeconomic status [2,7,11,12]. Several studies have demonstrated a rise in the mean systolic blood pressure (SBP) accompanying age increases in children [2,5,13-15]. One study reported that children with a SBP > 90^th ^percentile have a tendency to remain in the same percentile over time [16]. Gender differences in BP exist irrespective of age, race or other relevant factors [13,17,18]. Rosner et al. showed that the mean levels of SBP was higher in boys 12–14 years of age[19]. Other studies report higher BP levels in African American and Hispanic school-age children, when compared to Caucasian children [19-25]. Yet, other studies have reported no correlation between BP and ethnicity, particularly after adjusting for height, age and gender [15,18,26-29].

Obesity is a main effector of BP in children [14,15,25,30-33]. Hypertension, as well as dyslipidemia, type 2 diabetes, orthopedic problems, sleep apnea, and gall bladder disease, is one of many complications associated with obesity in children [34]. The percentage of obese children and adolescents has more than doubled since the early 1970s [35]. Obesity is also associated with development of acanthosis nigricans (AN), a skin lesion characterized by hyperpigmentation and a velvety thickening that occurs on the skin folds usually around the neckline. AN, as well as hypertension, are associated with hyperinsulinemia [36,37]. The increasing prevalence of obesity in children, the fact that BP in adulthood can be predicted by childhood and adolescent BP patterns, and that hypertension is one of the most important risk factors for cardiovascular disease; emphasizes the importance of assessing BP as a risk factor for hypertension and its complications as early as possible. The present study examined HBP in school-aged children and as well as the potential associations between demographic and physical factors.

## Methods

Data for this study was obtained from a previous study involving children from seventeen schools in Fort Worth, Texas. The schools were selected following a non-probabilistic sampling procedure developed by the investigators and the Independent School District (ISD) Director of Health Services in order to reflect the ethnic and geographical profile of the school district's student population. Of the total number of fifth graders (n = 1,500), 1,076 of those 8–13 years of age agreed to participate. Since there were missing values for 10 of these children, the final data set used in the analysis included 1,066 children.

Parents/guardians were provided with a description of the project, informed consent forms, and a family history/lifestyle questionnaire (written in both English and Spanish). If willing to allow their child to participate, parents/guardians were then asked to sign the informed consent form and complete the family history/lifestyle questionnaire. Children agreeing to participate signed the assent form. In addition, trained research assistants explained the study procedures and measurements to the participants. Information included in the dataset were age, date of birth, gender, ethnicity, height, weight, body mass index (BMI), systolic and diastolic blood pressure (SBP and DBP, respectively), and presence or absence of AN. Data were obtained from physical examination and completion of the questionnaire.

The classification of BP percentiles for this study was determined using normative tables generated from the National Health and Nutrition Examination Survey (NHANES) data submitted by the 1996 National High Blood Pressure Education Program Working Group on Hypertension Education in Children and Adolescents, which take into account the age, gender and height of each child [2]. The 95^th ^percentile was used to determine HBP for each child's age, gender, and height.

### Weight and Height

Children were weighed wearing light clothes and no shoes. Weight was recorded in pounds using a Tanita Model TBF-300 digital electronic scale. Height was recorded in inches to the nearest 1/16^th ^of an inch using a portable stadiometer. All measurements were recorded between 8:30 a.m. and 11:00 a.m. Weight and height were converted to metric measurements in order to determine the BMI, which is represented as weight (kg) divided by the square of height (m^2^). Children with a BMI value ≥ 85^th ^percentile for age and sex were classified as being overweight [38]. Overweight and obese categories were combined in this study and are described as obese in this paper.

### Acanthosis Nigricans (AN)

AN, as assessed by the research team pediatrician, was recorded as level 0–4, with 0 representing the absence of the condition [36,37]. Due to the small number of cases among the children, levels 1–4 were combined into aggregate categories of either "AN present" or "AN absent".

### Blood pressure (BP)

BP was measured after the child rested for at least 5 minutes in a sitting position. A registered nurse from the local Cook Children's Hospital performed BP measurements with an automated Dinamap 8100 XL monitor [39-41]. If the readings indicated that the BP was elevated or in the range for hypertension (90^th ^or 95th percentile, respectively, based on normative BP tables that take into account height, age and gender measured on at least three separate occasions), a second and third reading was taken after the child had rested for an additional 20 minutes. Since in this study BP was assessed on a single set of 3 measurements for all participants, to minimize misclassification average SBP and DBP was recorded[2]. According to the criterion set forth in the 1996 task force report on HBP in children and adolescents [2], in this study, hypertension in children was defined as average SBP or DBP ≥ 95^th ^percentile for age, sex, and height measured on at least three separate occasions. Elevated or high normal BP was defined as average SBP or DBP ≥ 90^th ^percentile, but less than the 95^th ^percentile [2]. Since BP was assessed using a single set of measurements, the 95^th ^percentile was used as the cut point to determine high BP in this study.

### Statistical Methods

Data analysis was performed using the SPSS statistical package (SPSS for Windows Version 11.5, 2002). The prevalence of SBP and DBP ≥ 95^th ^percentile in the study population, as well as the prevalence of isolated SBP and DBP ≥ 95^th ^percentile, were computed. Children with BP ≥ 95^th ^percentile were compared to children with BP < 95^th ^percentile regarding the association of potential covariables. The odds ratio was used as a measure of association. The crude associations were obtained for BP ≥ 95^th ^percentile with potential factors such as obesity. Multiple logistic regression analysis was used to assess the association of each covariable and BP ≥ 95^th ^percentile, adjusting for all potentially confounding variables simultaneously. To assess precision, the 95 % C.I. was calculated for crude and adjusted odds ratios [42,44].

## Results

The children in this study were 10–12 years of age. Although four children were outside of this age range (one 8, one 9, and two 13 year olds), they were not important outliers, therefore, they were added to the nearest single year age group. Due to the small number of children identified as Asian, or belonging to "other" ethnic/racial groups, these categories were not included in this study. Hispanics, African American, and Caucasians constituted 58.7 %, 24.6 %, and 16.7 % of the study participants, respectively. Approximately one-third of the participants were overweight. The prevalence of obesity was 32.8 %, 23.5 %, and 31.9 % in African American, Caucasian, and Hispanic children, respectively (data not shown). The prevalence of AN was 15.3 %. AN was diagnosed in 17 % and 21.8 % of Hispanic and African American children, respectively, however, it was absent in Caucasian children. For the purpose of this study, all children with BP ≥ 95th percentile, either isolated SBP or accompanied by DBP ≥ 95th percentile, were grouped into the general HBP category. Overall, the prevalence of HBP was 20.6 % (Table 1). The crude and adjusted odds ratios for the associations between HBP and demographic/physical characteristics are shown in Table 1. No difference was found by age group, yet slight differences were found for the age distribution between children with and without HBP. African Americans were 31 % more likely to have HBP than Caucasians. For Hispanics, the crude analysis showed a 71 % higher odds than Caucasians, however, this difference disappeared after adjustment. The likelihood of having HBP was at least 3 times higher among overweight children and among those with AN (Crude OR = 4.38; Adjusted OR = 3.05). After adjusting for confounding factors, the likelihood of having HBP was 3.05 times higher for overweight children (p < 0.001), and 2.36 times higher among those with AN (p < 0.001).

**Table 1:** Prevalence and Crude and Adjusted Odds Ratios of Hypertension^a ^by Demographic Variables in School-Age Children.

**Variable **	**Prevalence of Hypertension **^a ^**No. (%) **	**Total **	**Crude OR (95% CI) **	**Adjusted **^b^**OR (95% CI) **	**[p-value] **
*Age Group (years)*					
8–10	73 (19.6)	373	1.00	1.00	
11	122 (21.3)	572	1.11 (0.81–1.54)	0.87 (0.61–1.23)	[0.431]
12–13	15 (20.5)	73	1.06 (0.57–1.98)	0.92 (0.47–1.80)	[0.810]
Total	210 (20.6)	1018			
*Gender*					
Male	93 (18.9)	493	1.00	1.00	
Female	117 (22.3)	525	1.23 (0.91–1.67)	1.30 (0.93–1.81)	[0.120]
*Ethnicity*					
Caucasian	26 (15.3)	170	1.00	1.00	
African American	43 (17.2)	250	1.15 (0.68–1.96)	1.31 (0.74–2.33)	[0.355]
Hispanic	141 (23.6)	598	1.71 (1.08–2.70)	0.77 (0.48–1.26)	[0.297]
*BMI*					
Overweight/Obese	120 (38.3)	313	4.25 (3.10–5.84)	3.05 (2.11–4.41)	[<0.001]
Normal	90 (12.8)	705	1.00	1.00	
*Acanthosis Nigricans*					
AN (+)	70 (45.5)	154	4.34 (3.01–6.26)	2.36 (1.52–3.65)	[<0.001]
AN (-)	137 (16.1)	851	1.00	1.00	

^a ^Hypertension was determined using the normative BP tables for children and adolescents from the 1996 updated Task Force Report on High Blood Pressure in Children and Adolescents.^2^

^b ^Odds ratio adjusted for age, gender, ethnicity, obesity, and AN.

## Discussion

In this study, SBP > 95th percentile accounted for nearly all of the cases of BP > 95th percentile. The prevalence of BP > 95th percentile among children reported in earlier studies ranged from 1.2 % to 13 % [7-9]. However, in a more recent study that included mostly minority school children, the prevalence of BP > 95th percentile was 17 % at the first screening [11]. Similarly, in our study, 16 % of children had SBP > 95th percentile, (with or without DBP > 95th percentile), and 2 % had DBP > 95th percentile (with or without SBP > 95th percentile) at the first screening. The results of these studies suggest that BP > 95th percentile is not rare in children. Females showed a slightly higher risk of BP > 95th percentile than males (Adjusted OR = 1.30; CI: 0.93, 1.81).

In contrast to previous studies reporting higher BP levels in African American children when compared to their Caucasian counterparts [19-23], results of this study show that Hispanic children were more likely to have HBP than African American or Caucasian children. Previous studies have either compared Hispanics versus Caucasians, or African Americans versus Caucasians. In this study, however, these three groups were compared simultaneously. African American children showed slightly higher odds ratios when compared to Caucasian children. The crude analysis showed an increased likelihood for Hispanic (71%) children to have a BP ≥ 95^th ^percentile. Sorof et.al [11] compared these three ethnic groups simultaneously and reported a higher prevalence of BP ≥ 95th percentile among Hispanic children, at the first reading. Our findings may be influenced by the higher prevalence of obesity among the minority children in the sample [45,46]. However, the prevalence of obesity was slightly higher in African American than in Hispanic children (32.8 % and 31.9 %, respectively) in this sample.

Other hypertension studies that have evaluated socioeconomic status and stress in African American and Caucasian children, have found higher rates of HBP among African Americans[47,48]. These studies concluded that exposure to chronic environmental stress and low socioeconomic status contributes to hypertension among African American youth. Although our study did not evaluate socioeconomic status or stress, our study population was from the Fort Worth Independent School District, a school district with a large minority student population. Thus, it may be more homogenous in comparison to school districts in larger cities.

In this study, obesity was the most important identified factor affecting the BP distribution in this sample of children. This finding was consistent with other studies that evaluated BP in children [14,15,25,30-33,45]. Among all children in this study, being overweight increased the likelihood of hypertension over three times, after adjusting for age, gender and ethnicity.

Several factors may have influenced the prevalence of HBP, as well as the strength of the associations found in this study: 1) the high prevalence of obesity in this population; 2) use of the automated oscillometric instead of the auscultatory method^49 ^to measure BP; 3) family history of hypertension and socioeconomic status, were not available in this study; 4) the lower likelihood of HBP in African American children compared to white may be due to the smaller sample size of African American (n = 250) and Caucasian (n = 170) children as compared to Hispanic children (n = 598); and 5) because of the local nature of the study and the non-random sample, these findings cannot be generalized to the rest of the population; 6) since this was a cross-sectional study, exposures, disease, and/or outcome were assessed at a single point in time, therefore it is not possible to elucidate whether exposures preceded or resulted from the outcome variable[44,55,56].

There is controversy regarding the discrepancies between the oscillometric and auscultatory methods of BP measurements [50-54]. Weaver et al. showed that auscultatory SBP levels were 6.4 mm Hg lower, and DBP levels were 8.7 mm Hg higher than their oscillometric measurements[51]. Park et al. reported that both SBP and DBP levels were higher with the oscillometric method[51,53]. Conversely, O'Brien reported lower SBP and DBP levels with the automated monitor Dinamap[54]. Still, these findings were challenged in other studies which compared the accuracy of Dinamap monitors with BP measurements using invasive methods in adult, pediatric, and neonatal patients [40,50]. The Dinamap appears to be an accurate and reliable technique for non-invasive measurement of BP. It is simple to use, shows consistency over time, is more acceptable to children, and can be appropriate for BP screening [41]. In spite of these methodological issues, the findings of this study are consistent with recent studies that show HBP in children is increasing rapidly, especially among minority children. Sorof et. al. [11], using an automated oscillometric monitor, did show a prevalence of HBP ≥ 95^th ^percentile) at 19% in school children at the first screening.

Results of this study show that factors associated with HBP (e.g., obesity and ethnicity), may play a role in the development of hypertension at an early age, and can be used as an independent marker for increased likelihood of HBP in children. Studies show that weight and height in pre-adolescents and adolescents is consistently highly associated with BP and thereby, predicts hypertension in adult life[2,14,15,25,30-33,57,58]. Obesity, which is associated with metabolic problems such as hyperinsulinemia, hyperlipidemia, diabetes and hypertension in young adults and children [59-66] was strongly associated with HBP in this study. The constellation of risk factors in this study, namely, obesity and BP > 95th percentile, should be interpreted as early indications of risk for chronic diseases such as type 2 diabetes and cardiovascular disease, diseases more commonly seen in adults. These findings show the need to encourage health care providers to screen children for HBP, or ideally, to include BP measurement in the child's routine physical exam, especially those who are overweight [2]. Furthermore, broader recommendations stem from our findings. A more aggressive approach toward promoting healthy lifestyle practices is a necessity if we are to prevent childhood obesity. Examples of healthy lifestyle practices include increased physical activity, restrictions on television watching or playing video games, and avoiding food with a high fat and simple sugar content. These lifestyle changes should be implemented in all children as early in life as possible.

## Conclusion

Twenty one percent of school children had HBP, especially the prevalence was higher among the overweight and Hispanic group. The high prevalence of HBP among minority children, especially Hispanic children, suggests that the next generation of minorities, particularly Hispanic adults, will likely be at a higher risk of developing hypertension and associated chronic diseases. One of the most important concerns is the public health implications for U.S. future generations. These implications are critical for Hispanics since they are the fastest growing sector of the U.S. population [67]. Thus, BP monitoring and early diagnosis of hypertension in children is one of the best strategies for the prevention of chronic diseases in adulthood. The association identified here can be used as independent markers for increased likelihood of HBP in children. Health care providers should be prepared to play a key role in the prevention of obesity and its related risk factors for development of chronic diseases.

## Competing interests

The author(s) declare that they have no competing interests.

## Authors' contributions

XUR, CUE, SB: Participated in design of the study and helped to do statistical analysis and to write the manuscript.

JM, MB, PAR, KPS: Participated in the design of the study and helped to write manuscript.

All authors read and approved the final manuscript.

## Pre-publication history

The pre-publication history for this paper can be accessed here:


